# The impact of trade on employment: New evidence from a global value chains perspective

**DOI:** 10.1371/journal.pone.0285681

**Published:** 2023-09-28

**Authors:** Yue Mingyang, Yuan Hankun, Xu Chen, Jin Zhida

**Affiliations:** 1 Department of Economics, Jiangsu Administration Institute, Nanjing, China; 2 Post-Doctoral Research Center, Suzhou International Development Group, Suzhou, China; 3 Suzhou Financial Research Institute, Suzhou, China; 4 School of Economics, Wuhan University of Technology, Wuhan, China; Sri Lanka Institute of Information Technology, SRI LANKA

## Abstract

Given the magnitude impact of global value chains (GVCs) in reconstructing the pattern of world trade, its employment implications deserve thorough study. In this paper we explore the impact of GVCs position on employment across countries and its heterogeneous mechanisms. We perform an in-depth theoretical analysis followed by an empirical test using panel data for 56 industries in 42 countries from 2000 to 2014. The results show that enhancing the GVCs position will significantly increase employment, with a more pronounced effect in developing countries. Mechanical tests demonstrate a positive wage effect for developed countries. For developing countries, there is a positive demand effect and a negative factor substitution effect. Heterogeneity tests show that developed countries promote employment primarily by improving the forward GVCs position. Developing countries boost employment mainly by reducing the backward GVCs position. Further research has revealed that employment in developed countries has a clear preference for industries with higher GVCs, crowding out employment in other industries. This paper has enriched research on the employment implications of GVCs position and exploring the possible crowding effect during the evolution of the position of GVCs, which has been informative and insightful for countries in formulating GVCs participation and employment policies.

## 1. Introduction

The spread of industrial transfer in the world has deepened the global distribution of production links, and the global value chains (GVCs) have been formed. At the same time, the production process of products has evolved from a single production process in a single country to a new form that combines multiple countries and multiple production factors. The position in GVCs for exports of a country not only affects its profitability [1], but also has a fundamental impact on its domestic employment [2, 3]. The emergence of GVCs means that the links between trade and technology are becoming increasingly integrated. Producers use trade, investment and technology to maximize profits, leading to the fragmentation and expansion of international production and trade networks, which can have dramatic effects on labor markets [4]. Countries undertake different production links according to their comparative advantages and resource advantages. Therefore, it is increasingly important to explore the impact of trade on employment from the perspective of GVCs [5, 6].

Research on the relationship between trade and labor market has been going on for decades, but the existing literature focuses on the impact from traditional perspective, such as trade openness and liberalization [7, 8], especially in developing countries [9]. Some literature focuses on the impact of uncertainty policy and other shocks on employment [10, 11], and further dimensions of employment, such as skilled-unskilled employment and child labor [12, 13]. As GVCs have continuously strengthened ties between countries and industries, if only companies or industries engaged in import and export are considered when exploring the relationship between trade and employment, most information is overlooked [14]. Therefore, re-examining the relationship between trade and employment from the perspective of GVCs can not only evade the trap of total trade data [15], but also more comprehensively consider the indirect impacts of input-output relationships between industries and value chain links [1].

However, studies on the relationship between trade and employment from the perspective of GVCs are still in their infancy [14]. Wang et al. [16] examined the impact of the import competition in China on the results of the U.S. labor market from a value chain perspective. It was found that not only the direct import industries, but also the upstream suppliers and downstream customer industries of these industries were affected to varying degrees. Bacchetta and Stolzenburg [17] further pointed out that while supplier industries may be losses from import competition, they can switch from domestic suppliers to lower-priced foreign suppliers, so as to reduce costs, which can increase demand for their products and thus improve employment. Li et al. [18], Lv et al. [19] and Shi and Zhao [20] all studied the impact of China’s GVCs participation on employment from the enterprise level, and found that GVCs participation had a significant positive impact on employment. The literature above provides detailed empirical evidence for the impact of GVCs on employment, but they are all focused and sole country or influence factors, which shadows their applicability.

Based on the existing literature, this paper examines the relationship between trade and employment from the perspective of GVCs. The possible marginal contributions of this paper are mainly as follows:

First, we focus on the effect of changes in the position of GVCs on domestic employment, which is different from the existing literature that focuses on the degree of embedding in GVCs. Our strategy better describes the changing trend of a country’s demand for labor in terms of its improved international standing. Second, we describe the mechanism of GVCs position affects employment in three aspects: demand effect, wage effect and factor substitution effect. In addition, we analyze the heterogeneous effects of different evolutionary patterns on employment levels according to different evolutionary paths (forward and backward) of GVCs positions. Third, this paper is the first to empirically test using three-dimensional panel data from 56 industries in 42 major countries of the world from 2000 to 2014. Regressions were grouped according to the degree of country development (developed and developing) and industry category (manufacturing and services). In this way, the heterogeneous impact on employment caused by changes in the position of industries participating in GVCs in countries with different levels of development can be better demonstrated. Last but not least, we innovatively explore the possible crowding effect during the evolution of the position of GVCs. There is, to the best of our knowledge, no literature on the subject.

The paper continues as follows: In Section 2, we elaborate on the influencing mechanism from the theoretical perspective and puts forward the core hypotheses. Section 3 introduces the empirical model, indicator construction and data source sequentially. The results of the model are presented in Section 4. Robustness tests, mechanism tests, and grouping tests are also provided in this part. Lastly, Section 5 concludes.

## 2. Theoretical mechanisms and hypotheses

The position of the industry in GVCs is one of the key factors in determining the impact of participation in GVCs on employment. Moreover, the employment implications of industry GVCs’ position are far from straightforward. Therefore, we perform the mechanism analysis from three perspectives, including demand effect, wage effect and factor substitution effect.

The first is the demand effect. The position of GVCs measures the position and length of an industry in the industrial chain [21]. The improvement of the position of GVCs is predominantly achieved through the improvement of the position of the industry chain (that is, the increase in the proportion of intermediate product exports in total exports) or the extension of the industry chain (the final product contains fewer intermediate products from other countries). The former mainly relies on research and development and production of new products with higher levels of technology, which will increase the demand for high-tech workers. The latter refers to the increasing improvement of the domestic industrial chain and the substitution of imported intermediate products, which will result in the transfer of foreign jobs to the country, driving up domestic employment levels. In general, an improvement in the position of GVCs is usually accompanied by an increase in the size of production, which inevitably has a positive impact on labor demand.

The second is the wage effect. Labor wages are an important factor in employment, and an improvement in the position of GVCs will affect wage levels and therefore employment levels [8]. On the one hand, the integration of GVCs, like skill-biased technological advances, has the potential to have a significant impact on the relative demand for skilled workers in an economy. This will increase the wages of the middle and high-skilled labor, and the increase in wages will further boost employment, creating a positive cycle [3]. On the other hand, however, if labour wages climb, this could also lead to an increase in the cost of production for companies, and thus to a sifting out of employment of some low-skilled workers, especially in labour-intensive production links at the lower end of the value chain.

The third is the factor substitution effect. Both capital and labor are essential elements of production, and there is a complementarity and a certain substitution between them. This paper explores two aspects, namely the total amount of capital and the proportion of capital factor returns. First, improving the position of GVCs would make the industry more lucrative, which would attract a large influx of domestic and foreign capital; In addition, an increase in the stock of fixed capital will boost employment, such as the creation of new production links. However, it can also have a substitution effect on employment. In order to satisfy stricter quality standards, it usually leads to expanded mechanization of firms, which can crowd out jobs [22]. Second, the fraction of factor income remunerated will alter the fraction of factor input. When the proportion of remuneration paid to the capital factor increases, it means that the income of the capital factor is greater than that of the labor factor, and this will stimulate the producer to invest more capital, and thus have a substitution effect on the labor, and thus reduce employment.

On the basis of the above analysis, the following two core hypotheses are proposed in the paper to be verified:

Hypothesis 1: provided other conditions remain consistent, an industry’s rise in GVCs position will increase its employment level.
Hypothesis 2: provided other conditions remain consistent, the changes of GVCs position will affect the employment level of the industry through demand effects, wage effects and factor substitution effects.

## 3. Empirical model, indicator construction and data source

### 3.1 Empirical model

The primary aim of this paper is to investigate the impact of GVCs position on employment. Based on the above theoretical analysis, the regression model is built as follows:

$$\ln L_{ijt}=a_{0}+\alpha_{1}GVCP_{ijt}+a_{2}\ln G_{ijt}+a_{3}\ln K_{ijt}+\alpha_{4}\ln W_{ijt}+a_{5}\ln cl_{ijt}+a_{6}\ln TE_{ijt}+a_{7}open_{ijt}+\mu_{ij}+\mu_{t}+\varepsilon_{ijt}$$ (1)

In this formula, *i* is the country; *j* is the industry; *t* is the year; *L*_*ijt*_ is the labor force employment in the *j* industry of *i* country during the *t* period; *GVCP*_*ijt*_ is the global value chain position index of the *i* country *j* industry in the t period. Given that other factors may have an impact on employment, following Jin et al. [1], we introduce control variables. Among them, ln *G*_*ijt*_ is the total output value (industry development scale); ln *K*_*ijt*_ is the fixed capital stock (capital deepening); ln *W*_*ijt*_ is the labor wage level (labor price); ln *cl*_*ijt*_ is the proportion of capital labor factor remuneration (reflecting the relative position of labor and capital in income distribution); ln *TE*_*ijt*_ is the total energy consumption; *open*_*ijt*_ is the share ratio of industry openness (trade openness, the percentage of total exports of the bank in total output) of the manufacturing industry of i country in period t respectively; *μ*_*ij*_ is the fixed effect multiplied by country and industry, *μ*_*t*_ is the time fixed effect and *ε*_*ijt*_ is the random error term.

To further test the mechanism of the influence of GVCs position changes on employment, the moderation model is adopted to test Hypothesis 2. Following Erik (2019), we build the following regression model:

$$\begin{array}{l} \ln L_{ijt}=a_{0}+\alpha_{1}GVCP_{ijt}+\beta_{1}(GVCP_{ijt}\times \ln G_{ijt})+\beta_{2}(GVCP_{ijt}\times \ln W_{ijt})\\ \;+\beta_{3}(GVCP_{ijt}\times \ln K_{ijt})+\beta_{4}(GVCP_{ijt}\times \ln cl_{ijt})+a_{2}\ln G_{ijt}+a_{3}\ln K_{ijt}\\ \;+\alpha_{4}\ln W_{ijt}+a_{5}\ln cl_{ijt}+a_{6}\ln TE_{ijt}+a_{7}open_{ijt}+\mu_{ij}+\mu_{t}+\varepsilon_{ijt} \end{array}$$ (2)

### 3.2 Construction of global value chains position *(GVCP)* indicators

Following Koopman et al. [23], *GVCP* is defined as the log ratio of a country industry’s supply of intermediates used in other countries’ exports to the use of imported intermediates in its own production. We further subdivided *GVCP* into forward GVCs position index (GVCPf) and backward GVCs position index (GVCPb). The mathematical expression can be deduced as follows:

$$GVCP^{ir}=GVCPF-GVCPB=\mathrm{Ln}(1+\frac{IV^{ir}}{uE^{ir}})-\mathrm{Ln}(1+\frac{FV^{ir}}{uE^{ir}})$$ (3)

To further resolve the double-counting problem, we adopt the value-added decomposition method proposed by Koopman et al. [15] to decompose the total export value-added of a country into nine parts:

$$\begin{array}{l} uE^{i*}=V^{i}\sum_{j\neq i}B^{ii}Y^{ij}+V^{i}\sum_{j\neq i}B^{ij}Y^{jj}+V^{i}\sum_{j\neq i}\sum_{t\neq i,j}B^{ij}Y^{jt}+V^{i}\sum_{j\neq i}B^{ij}Y^{ji}+V^{i}\sum_{j\neq i}B^{ij}A^{ji}(I-A^{ii}{)}^{-1}Y^{ii}\\ +V^{i}\sum_{j\neq i}B^{ij}A^{ji}(I-A^{ii}{)}^{-1}E^{i*}+\sum_{t\neq i}\sum_{j\neq i}V^{t}B^{ti}Y^{ij}+\sum_{t\neq i}\sum_{j\neq i}V^{t}B^{ti}A^{ij}(I-A^{jj}{)}^{-1}Y^{jj}+\sum_{t\neq i}V^{t}B^{ti}A^{ij}\sum_{j\neq i}(I-A^{jj}{)}^{-1}E^{j*} \end{array}$$ (4)

Combining Eqs (3) and (4), *uE*^*i*^_*r*_ means the gross exports of country *i*’s *r* industry. $IV_{r}^{i}=V^{i}\sum_{j\neq i}\sum_{t\neq i,r}B^{ij}Y^{jt}$, is the domestic value-added in intermediate products of industry *r* in country *i* re-exported to third countries. $FV_{r}^{i}=\sum_{t\neq i}\sum_{j\neq i}V^{t}B^{ti}Y^{ij}+\sum_{t\neq i}\sum_{j\neq i}V^{t}B^{ti}A^{ij}{\left(I-A^{jj}\right)}^{-1}Y^{jj}$, represents the total foreign value-added contained in the country *i*’s *r* industry [21].

### 3.3 Data sources and statistical description

We adopt the World Input-Output Database (WIOD) released by the European Commission in 2016. The database contains two sub-databases: WIOT and Socio-economic Account. The WIOD database provides a continuous WIOT series with 15-year (2000–2014), containing 56 industries in 44 countries or regions (28 EU members and 15 major economies and the rest of the world (ROW)) and covering all industry categories in the International Standard Industrial Classification, Revision 4 (ISIC Rev 4.0). The Socio-economic Account mainly contains data on output, prices, capital stock and employment. The energy consumption data in this paper comes from the WIOD energy account published by the European Commission in 2019. This paper organizes the database and obtains panel data of 56 industries in 42 major countries from 2000 to 2014. The descriptive statistics of the main variables are shown in Table 1.

**Table 1:** Statistics of main variables.

	Total Sample			Developed Countries			Developing Country		
**Variable**	Obs	Mean	Std.Dev.	Obs	Mean	Std.Dev.	Obs	Mean	Std.Dev.
**Ln*emp***	33246	4.078	2.3	21879	3.825	2.104	11367	4.566	2.569
***GVCP***	35280	-0.156	0.166	22680	-0.151	0.15	12600	-0.166	0.191
***GVCPf***	35280	0.069	0.035	22680	0.07	0.033	12600	0.067	0.038
***GVCPb***	35280	0.225	0.144	22680	0.221	0.131	12600	0.232	0.164
**Ln*G***	33249	9.797	3.3	21879	9.575	2.982	11370	10.225	3.803
**Ln*K***	32799	9.587	3.441	21541	9.344	3.118	11258	10.052	3.946
**Ln*W***	33245	4.08	2.011	21879	4.182	1.797	11366	3.884	2.357
**Ln*cl***	31636	-0.374	1.134	20694	-0.508	1.11	10942	-0.121	1.136
**Ln*TE***	32834	9.023	2.825	21609	8.998	2.813	11225	9.071	2.847
***Open***	33249	24.075	27.105	21879	25.347	27.79	11370	21.626	25.56
***GVCPIV***	32954	0.304	0.138	21734	0.325	0.146	11220	0.263	0.112

## 4. Empirical results

### 4.1 Benchmark regression results

To empirically investigate the impact of the evolution of the position of GVCs on the employment levels of different industries in various countries, we conduct an empirical analysis using panel data for 56 industries in 42 major countries of the world from 2000 to 2014. We adopt a three-dimensional fixed effects model, control the time fixed effects and the fixed effects of country* industry. At the same time, this paper is clustered to country* industry.

Table 2 reports the regression results for the effect of the GVCs position on the employment level of the sector when the control variables are added sequentially. The results show that the coefficient of the GVCs position is always positive and statistically significant at the 1% critical level, indicating that there is a strong positive correlation between the GVCs position and employment in the sector. When the control variables are added sequentially, the results remain stable. This shows that improving the position of GVCs will indeed increase employment in the sector. As a result, Hypothesis 1 is well verified.

**Table 2:** Benchmark regression results.

	(1)	(2)	(3)	(4)	(5)	(6)	(7)
***GVCP***	1.016***	0.487***	0.490***	0.345***	0.808***	0.818***	0.848***
	(0.2218)	(0.1050)	(0.1076)	(0.0865)	(0.0639)	(0.0641)	(0.0648)
**Ln*G***		0.342***	0.320***	0.484***	0.571***	0.554***	0.551***
		(0.0159)	(0.0212)	(0.0232)	(0.0179)	(0.0181)	(0.0181)
**Ln*K***			0.0273	0.149***	0.161***	0.161***	0.162***
			(0.0177)	(0.0193)	(0.0135)	(0.0135)	(0.0135)
**Ln*W***				-0.585***	-0.723***	-0.713***	-0.712***
				(0.0232)	(0.0159)	(0.0160)	(0.0160)
**Ln*cl***					-0.151***	-0.149***	-0.149***
					(0.0064)	(0.0063)	(0.0063)
**Ln*TE***						0.024***	0.023***
						(0.0047)	(0.0047)
***Open***							-0.001***
							(0.0003)
**_cons**	4.222***	0.800***	0.751***	0.344*	-0.063	-0.149	-0.085
	(0.0314)	(0.1596)	(0.1661)	(0.1372)	(0.1143)	(0.1159)	(0.1177)
country* industry	Y	Y	Y	Y	Y	Y	Y
**year**	Y	Y	Y	Y	Y	Y	Y
**N**	33246	33246	32796	32796	31483	31242	31242
**adj.R-sq**	0.9917	0.9933	0.9934	0.9958	0.9972	0.9973	0.9973

Notes: Standard errors are calculated by clustering over country*industry level and reported in parentheses.

***p < 0.01

**p < 0.05

*p < 0.1.

Among the control variables, the coefficients of total output value, fixed capital stock and total energy consumption are positive, which indicates that there is a significant scale effect of expanding production scale on employment. However, the coefficients of wage level, proportion of capital labor factor remuneration and industrial openness are negative. This may be owing to the fact that capital factor has a certain substitution effect on labor. The improvement of opening-up may lead to a large number of enterprises to conduct outsourcing activities and transfer part of domestic employment to foreign countries [24].

### 4.2 Endogeneity treatment and 2SLS estimation

The endogeneity problem arising from the potential mutual causality between GVCs position and employment cannot be ignored. In this subsection, we re-evaluate the model using 2SLS estimation by constructing various instrumental variables. First, according to Jin et al. [1], we construct instrumental variables for GVCs position. Specifically, we take the ratio of the double-counted intermediate exports produced abroad to the foreign content as the instrumental variable of GVCs position, designated as GVCIV. Second, we also use the one and two lag periods of the GVCP as its instrumental variables for regression, denoted as *L*.*GVCP* and *L2*.*GVCP*, respectively. The results are reported in Table 3.

**Table 3:** Results of the endogeneity treatment and 2SLS estimation.

	(1)	(2)	(3)
***GVCP***	0.977**	0.791***	0.840***
	(0.3728)	(0.0771)	(0.1321)
**Ln*G***	0.546***	0.545***	0.523***
	(0.0216)	(0.0188)	(0.0198)
**Ln*K***	0.160***	0.167***	0.180***
	(0.0138)	(0.0145)	(0.0141)
**Ln*W***	-0.712***	-0.714***	-0.712***
	(0.0159)	(0.0155)	(0.0158)
**Ln*cl***	-0.151***	-0.144***	-0.142***
	(0.0085)	(0.0063)	(0.0068)
**Ln*TE***	0.024***	0.023***	0.024***
	(0.0049)	(0.0049)	(0.0050)
***Open***	-0.001***	-0.001***	-0.001**
	(0.0004)	(0.0003)	(0.0003)
country*industry	Y	Y	Y
**Year**	Y	Y	Y
**N**	31242	29168	27077
**First stage**			
***GVCPIV***	0.409*** (0.0531)		
***L*.*GVCP***		0.711*** (0.0110)	
***L2*.*GVCP***			0.381*** (0.0772)
**F-statistics**	59.28	4173.13	24.35

Notes: Standard errors are reported in parentheses.

***p < 0.01

**p < 0.05

*p < 0.1.

Column (1) in Table 3 reports the regression results of GVCIV as the instrumental variable of GVCs position. Columns (2)–(3) represent the regression results of one and two lagging periods of GVCP as its instrumental variable. As shown in the first-stage regression, the coefficients of the instrumental variables are all significant at the 1% critical level, implying that instrumental variables are strongly correlated with explanatory variables. In addition, the large Kleibergen-Paap Wald rk F statistics show that the instruments are robust [1]. The coefficients of the key term *GVCP* are still positive and significant, even larger than the magnitude of baseline regression results. This shows that it is relatively robust for benchmark regression results.

### 4.3 Group regression results

The deepening of GVCs is a process of cross-country and cross-sector resource integration. Due to great differences in resource endowments and industry development stages across countries, GVCs shocks will also have heterogeneous impacts on employment levels and structures [25, 26]. The emergence of GVCs has provided new opportunities for developing countries to integrate into the global economy. At the same time, some studies have found that there are significant differences in the changes in labor demand caused by trade in the manufacturing and service industries [3, 24]. Therefore, we perform group regression to further investigate the relationship between the position of GVCs and employment in economies with different levels of development and different types of industries. The results are shown in Table 4.

**Table 4:** Group regression results.

	(1)	(2)	(3)	(4)	(5)	(6)
	Developed Country			Developing Country		
	Total Sample	Manufacturing	Service	Total Sample	Manufacturing	Service
***GVCP***	0.809***	1.159***	0.553***	0.947***	1.197***	0.728***
	(0.0722)	(0.1296)	(0.0937)	(0.1222)	(0.1962)	(0.1575)
**Ln*G***	0.514***	0.476***	0.499***	0.567***	0.472***	0.663***
	(0.0206)	(0.0249)	(0.0360)	(0.0340)	(0.0529)	(0.0474)
**Ln*K***	0.157***	0.161***	0.118***	0.159***	0.245***	0.106***
	(0.0180)	(0.0267)	(0.0235)	(0.0200)	(0.0327)	(0.0240)
**Ln*W***	-0.549***	-0.566***	-0.483***	-0.810***	-0.801***	-0.832***
	(0.0285)	(0.0594)	(0.0419)	(0.0190)	(0.0273)	(0.0247)
**Ln*cl***	-0.119***	-0.124***	-0.102***	-0.194***	-0.194***	-0.203***
	(0.0066)	(0.0125)	(0.0080)	(0.0116)	(0.0184)	(0.0150)
**Ln*TE***	0.016***	0.024***	0.006	0.050***	0.020	0.042
	(0.0045)	(0.0063)	(0.0080)	(0.0138)	(0.0152)	(0.0267)
***Open***	-0.001*	-0.001**	-0.000	-0.002***	-0.001	-0.001
	(0.0004)	(0.0004)	(0.0007)	(0.0005)	(0.0007)	(0.0010)
**_cons**	-0.345	-0.183	0.235	0.012	0.219	-0.156
	(0.1763)	(0.2464)	(0.3092)	(0.2353)	(0.3443)	(0.3099)
country*industry	Y	Y	Y	Y	Y	Y
**year**	Y	Y	Y	Y	Y	Y
**N**	20451	7382	10132	10791	3977	5214
**adj.R-sq**	0.9974	0.9972	0.9978	0.9972	0.997	0.9973

Notes: Standard errors are calculated by clustering over country*industry level and reported in parentheses.

***p < 0.01

**p < 0.05

*p < 0.1.

In Table 4, Columns (1)—(3) list the samples of developed countries, which are regression results of the whole sample, manufacturing and service samples, respectively; Columns (4)—(6) are similar to the first three columns, except that they are samples of developing countries. It is found that for the overall sample, the coefficient of the GVCP is always positive at the 1% critical level. In addition, the coefficients of developing countries are slightly higher than those of developed countries. This indicates that the improvement in GVCP in developing countries plays a larger role in the growth of domestic employment. The possible reason is that compared with developed countries, developing countries tend to have abundant labor resources and lower wages, which makes employment levels increase rapidly during the process of GVCs participation [22, 27].

In terms of different industries, the GVCP coefficient of manufacturing is much higher than that of services. This demonstrates that trade does make a big difference in the employment levels of different industries. This may be due to the greater demand for labor in manufacturing than in services, particularly in developing countries, which have large numbers of unskilled labor and tend to be at the lower end of the GVCs. GVCs provide a channel for developed countries to disseminate new technologies to developing countries, which will generate more learning spillovers and help enhance the level and quality of employment in developing countries [27]. An interesting phenomenon is that the coefficients of manufacturing industries in developing countries are close to those in developed countries, but the difference between the coefficients of service industries in developing countries and in developed countries is significantly larger. The reason may be that the developed world’s service sector is relatively well-supplied, while the developing world’s service sector is largely in a period of rapid development and has a relatively large marginal demand for labor.

### 4.4 Mechanism test

In this subsection, we empirically test the theoretical mechanism proposed in Section 2. We first performed a group regression analysis using the moderated model, and the results are presented in Table 5. We find that, in general, GVCP affects employment mainly through positive demand effects and negative factor substitution, while wage effects are negligible, in agreement with the expectations of Hypothesis 2. In terms of grouping, developed countries only show a significant positive wage effect in the services sector, which may be a result of the continuous adjustment of industrial structure during participation in GVCs. The continuous development of the service industry makes the service economy become the leading force in the world economy [28]. Developed countries have advanced service industries, and the continuous improvement of the GVCP will promote the continuous improvement of wages and further attract high-tech talent to join, forming a virtuous circle. In contrast, developed countries have better manufacturing infrastructure and higher levels of mechanization, so expanding production scale and additional capital investment do not stimulate employment as well. This is in line with current reality.

**Table 5:** Mechanism test results of scale effect, cost effect and factor substitution effect.

	(1)	(2)	(3)	(4)	(5)	(6)	(7)
	Total Sample	Developed Country			Developing Country		
		Total Sample	Manufacturing	Service	Total Sample	Manufacturing	Service
***GVCP***	0.850***	0.866***	1.000**	0.867**	0.891***	0.364	1.003**
	(0.1749)	(0.2153)	(0.3471)	(0.3169)	(0.2684)	(0.4146)	(0.3496)
***GVCPG***	0.177**	-0.005	-0.002	-0.144	0.360***	0.462*	0.231*
	(0.0663)	(0.0751)	(0.1065)	(0.1115)	(0.1028)	(0.1804)	(0.1059)
***GVCPW***	0.0527	0.173**	0.181	0.215*	-0.024	-0.080	-0.052
	(0.0435)	(0.0611)	(0.1131)	(0.0844)	(0.0559)	(0.0919)	(0.0743)
***GVCPK***	-0.209***	-0.085	-0.071	0.005	-0.355***	-0.378**	-0.236**
	(0.0578)	(0.0696)	(0.1082)	(0.0923)	(0.0845)	(0.1442)	(0.0877)
***GVCPcl***	-0.083*	-0.037	0.021	-0.081	-0.081	-0.203*	-0.092
	(0.0362)	(0.0409)	(0.0834)	(0.0455)	(0.0558)	(0.0794)	(0.0723)
**Ln*G***	0.586***	0.513***	0.477***	0.471***	0.629***	0.558***	0.698***
	(0.0173)	(0.0209)	(0.0330)	(0.0331)	(0.0278)	(0.0356)	(0.0464)
**Ln*K***	0.122***	0.138***	0.144***	0.116***	0.109***	0.185***	0.078**
	(0.0136)	(0.0169)	(0.0311)	(0.0203)	(0.0195)	(0.0275)	(0.0260)
**Ln*W***	-0.707***	-0.522***	-0.535***	-0.450***	-0.818***	-0.818***	-0.843***
	(0.0176)	(0.0282)	(0.0620)	(0.0404)	(0.0214)	(0.0329)	(0.0264)
**Ln*cl***	-0.166***	-0.127***	-0.120***	-0.117***	-0.210***	-0.226***	-0.223***
	(0.0089)	(0.0090)	(0.0183)	(0.0109)	(0.0157)	(0.0255)	(0.0200)
**Ln*TE***	0.023***	0.017***	0.025***	0.004	0.046***	0.021	0.037
	(0.0047)	(0.0045)	(0.0064)	(0.0080)	(0.0134)	(0.0146)	(0.0252)
***Open***	-0.001***	-0.001*	-0.001**	-0.000	-0.002***	-0.001	-0.001
	(0.0003)	(0.0004)	(0.0004)	(0.0007)	(0.0005)	(0.0006)	(0.0009)
**_cons**	-0.080	-0.272	-0.17	0.391	-0.047	-0.048	-0.137
	(0.1131)	(0.1767)	(0.2362)	(0.3053)	(0.2015)	(0.2599)	(0.2875)
country* industry	Y	Y	Y	Y	Y	Y	Y
**year**	Y	Y	Y	Y	Y	Y	Y
**N**	31242	20451	7382	10132	10791	3977	5214
**adj. R-sq**	0.9973	0.9975	0.9972	0.9979	0.9972	0.9971	0.9973

Notes: Standard errors are calculated by clustering over country*industry level and reported in parentheses.

***p < 0.01

**p < 0.05

*p < 0.1.

For developing countries, there are significant positive scaling effects and significant negative factor substitution effects. The former is due to the fact that most developing countries are still at the low end of the GVCs and are mainly engaged in labor-intensive production activities, whose scale-up will inevitably lead to a significant increase in labor demand. The latter is because developing countries may enjoy technology spillover effects from developed countries in the process of participating in GVCs. As a result, when developing countries upgrade their production processes and plug in advanced production machines, they will have a substantial spillover effect on the less skilled workforce [9]. This is even more evident in the manufacturing sector, as the return to capital factor in manufacturing in developing countries also indicates a significant negative correlation.

### 4.5 Heterogeneous analysis

In this subsection, we further explore the heterogeneous impact of the position of GVCs on employment from different evolutionary paths. Panels A and B in Table 6 show the heterogeneous impact of the positions of forward and backward GVCs on employment, respectively. It is found that the coefficient of the forward GVCP is significantly positive and the coefficient of the backward GVCP is significantly negative, which is consistent with the expectations of hypothesis 3. For developed countries, the coefficient of the forward GVCP is significantly larger than that of the backward GVCP, indicating that developed countries mainly promote employment by promoting the forward GVCP. This is because most developed countries occupy the upper part of the value chain of various industries. In forming a global production network, they retain high-tech production links and outsource low-end production links of the industrial chain to less developed regions. Thus, the forward GVCP has a larger impact on employment than the backward GVCP.

**Table 6:** Results of the effect of forward and backward GVCs position on employment.

	(1)	(2)	(3)	(4)	(5)	(6)	(7)
**Effect of forward GVCs position on employment**							
***GVCPf***	1.486***	1.483***	2.152***	0.992*	1.459***	1.530**	1.343*
	(0.2354)	(0.2913)	(0.4478)	(0.4154)	(0.3926)	(0.5588)	(0.5189)
**_cons**	-0.613***	-0.899***	-0.800**	-0.179	-0.713***	-0.484	-0.785**
	(0.1143)	(0.1804)	(0.2724)	(0.3069)	(0.2103)	(0.3338)	(0.2646)
**Effect of backward GVCs position on employment**							
***GVCPb***	-1.067***	-1.011***	-1.467***	-0.694***	-1.209***	-1.633***	-0.886***
	(0.0786)	(0.0876)	(0.1647)	(0.1081)	(0.1469)	(0.2376)	(0.1863)
**_cons**	0.075	-0.206	-0.002	0.342	0.226	0.509	-0.014
	(0.1202)	(0.1760)	(0.2452)	(0.3091)	(0.2440)	(0.3542)	(0.3213)
**Control variable**	Y	Y	Y	Y	Y	Y	Y
country* industry	Y	Y	Y	Y	Y	Y	Y
**year**	Y	Y	Y	Y	Y	Y	Y
**N**	31242	20451	7382	10132	10791	3977	5214
**adj.R-sq**	0.9971	0.9973	0.9969	0.9978	0.997	0.9967	0.9972

Notes: Standard errors are calculated by clustering over country*industry level and reported in parentheses.

***p < 0.01

**p < 0.05

*p < 0.1.

For developing countries, the backward GVCP coefficient is larger for manufacturing, while the forward GVCP coefficient is larger for services. The former is due to the fact that manufacturing in developing countries is mainly at the lower end of the value chain. In the process of improving its GVC position, it mainly aims to reduce its backward GVC position by producing intermediate products through independent innovation and continuously improving its independent industrial chain. That would boost employment. The latter may be due to the continuous development and improvement of the service sector in developing countries in recent years and its increasing role in promoting employment. The latter may be due to the continuous development and improvement of the service industry in developing countries in recent years, and its increasing role in promoting employment.

### 4.6 Further discussion

Previous studies have shown that trade will lead to international employment transfer [29, 30]. However, from the perspective of GVCs, there is little literature on employment transfer between different industries in a country. In this subsection, we perform a preliminary study of this problem. First, we study the relationship between the GVCP and the share of its employment in the total employment of a country’s industries. I.e., whether there is a preference for employment in industries with high GVCP. Second, we explore the impact of changes in the GVCP on the share of total employment in a country’s industries. I.e., whether changes in the GVCP of this sector crowd out employment in other sectors. The results are shown in panels A and B of Table 7, respectively.

**Table 7:** Effect of GVCs position on the proportion of employment in the industry.

	(1)	(2)	(3)	(4)	(5)	(6)	(7)
**Panel A: Effect of GVCs position on the proportion of employment in the industry**							
***GVCP***	0.009***	0.011***	0.007***	0.013**	0.003	0.003	0.002
	(0.0018)	(0.0025)	(0.0016)	(0.0046)	(0.0027)	(0.0017)	(0.0037)
**_cons**	-0.011***	-0.017***	-0.012***	-0.013	-0.023***	-0.030***	-0.010
	(0.0031)	(0.0039)	(0.0034)	(0.0069)	(0.0052)	(0.0059)	(0.0058)
**N**	31242	20451	7382	10132	10791	3977	5214
**adj.R-sq**	0.9844	0.9908	0.9655	0.9926	0.9787	0.9655	0.9835
**Panel B: Effect of changes in GVCs position on the proportion of employed in the industry**							
***D*.*GVCP***	0.006***	0.007***	0.004**	0.009***	0.005***	0.005***	0.006**
	(0.0009)	(0.0014)	(0.0014)	(0.0021)	(0.0011)	(0.0008)	(0.0021)
**_cons**	-0.000	-0.000	-0.000***	0.000*	-0.000	-0.000***	0.000***
	(0.0000)	(0.0000)	(0.0000)	(0.0000)	(0.0001)	(0.0000)	(0.0000)
**N**	28646	18732	6788	9258	9914	3665	4783
**adj.R-sq**	0.213	0.2321	0.1815	0.259	0.2127	0.2623	0.1554
**Control variable**	Y	Y	Y	Y	Y	Y	Y
country* industry	Y	Y	Y	Y	Y	Y	Y
**year**	Y	Y	Y	Y	Y	Y	Y

Notes: Standard errors are calculated by clustering over country*industry level and reported in parentheses.

***p < 0.01

**p < 0.05

*p < 0.1.

Panel A of Table 7 illustrates the regression results for the GVCP and the proportion of employment in the sector. It was found that there was a significant positive relationship between employment levels and GVCP only in developed countries, but not in developing countries. In terms of industry categories, manufacturing and service industries in developed countries show significant positive correlations. This indicates that the workforce in developed countries prefers higher positions in GVCs. This may be due to the higher human capital of the workforce in developed countries. However, industries with higher levels of the global value chain have a higher demand for high-tech labor and higher wages, which will attract high-tech talent. This is also consistent with the significant wage effect in the mechanistic tests of Table 6.

Panel B of Table 7 shows the results of the regression of the GVCP change on the proportion of people employed in the sector, thus reflecting the adjustment of the employment structure at the national level. We find that, in general, the promotion of GVCP in both developed and developing countries will significantly increase the share of employment in this sector, with larger coefficients in developed countries. In terms of industry, the coefficient of the service sector is higher than that of the manufacturing sector, which is more pronounced in developed countries. The coefficient for services in the developed world is more than twice that of manufacturing, suggesting that changes in the GVCP of services in the developed world have a more significant impact on their employment structure. This could be one explanation for the hollowing out of manufacturing in the developed world. On the one hand, developed countries have shifted production links at the lower end of the value chain overseas through outsourcing, which has led to a decline in domestic manufacturing employment. On the other hand, some manufacturing jobs have been squeezed out by the rapid development of domestic services.

## 5. Conclusion and policy implications

The deepening of GVCs has reshaped the international trade landscape and has exerted a giant influence on the development of the world economy. However, there is still a gap in studying the impact of trade on employment from the perspective of GVCs. In this paper, we discuss the mechanisms by which the GVCs position affects employment, and present a detailed empirical analysis based on data from 56 industries in 42 major countries from 2000 to 2014.

The empirical results demonstrate that: (1) there is a significant positive correlation between GVCs position and employment. (2) Group regression shows that the enhancement of GVCs position in developing countries plays a greater role in promoting employment. Manufacturing has a considerably higher coefficient than services. (3) Mechanism test illustrates that there is only a significant positive wage effect in the service industry for developed countries, while the developing countries have a significant positive demand effect and a negative factor substitution effect. In addition, developed countries primarily promote employment by increasing the position of forward GVCs, while developing countries primarily promote employment by decreasing the position of backward GVCs. (4) Further research reveals that the labor force of developed countries has a significant preference for the industries with a higher position in the GVCs. Improvements in the position of GVCs will increase the proportion of employment in that sector, that is, improvements in the position of GVCs in one sector may have a crowding-out effect on employment in other sectors.

The research presented in this paper has strong policy implications for countries participating in international trade, promoting domestic employment, and achieving high-quality economic development in the era of GVCs. First, countries should continuously strive to improve their position in the process of participating in GVCs. In particular, developing countries need to encourage innovation to achieve technological progress and industrial upgrading, so as to break through the dilemma of "low-end lock-in". Our study confirms that the promotion of GVCs positions can not only bring greater benefits to international trade, but also further boost employment. Second, from the perspective of the evolution of GVCs, developing countries mainly promote employment by reducing the position of backward GVCs, i.e., by improving and upgrading the industrial chain. This suggests that developing countries should further improve the construction of domestic circulation and enhance the position of forward GVCs to better align with current domestic and international development trends. Third, as industries with high positions in the GVCs of developed countries are attractive for employment, especially services, developed countries should rationally guide employment and avoid excessive hollowing out of manufacturing.

Some limitations of this paper include that the accuracy of the results depends on the availability of data, by period and by country. However, this paper explores the impact of GVCs position on employment in depth, and provides guidance and references for related policies. When data is available, a more comprehensive field study could cover more countries and over a longer period of time, while further breaking down employment into skill levels.
